# Immune monitoring in patients with colorectal cancer stage II

**DOI:** 10.1186/2051-1426-3-S2-P266

**Published:** 2015-11-04

**Authors:** Eva Zavadova, Michal Vocka, Jan Spacek, Nicole Francis, Bohuslav Konopasek, Terezie Fucikova, Helena Skalova, Pavel Dundr, Lubos Petruzelka, Tilman T Rau, Carol Geppert, Arndt Hartmann

**Affiliations:** 1 Department of Oncology, General Teaching Hospital and 1st Faculty of Medicine Charles University, Prague, Czech Republic; 2 1st Faculty of Medicine Charles University, Prague, Czech Republic; 3 Institute of immunology and microbiology, General Teaching Hospital and 1st Faculty of Medicine Charles University, Prague, Czech Republic; 4 Dept. of pathology, General Teaching Hospital and 1st Faculty of Medicine Charles University, Prague, Czech Republic; 5 Institute of Pathology, University Erlangen-Nürnberg, Erlangen, Germany

## 

Immunoscore has been shown to be a very powerful prognositc indicator in patients with clinically localized colorectal cancer, with no detectable tumour spread to lymph nodes or distant organs. These patients are usually treated with surgical removal of the tumour only. However, approximately 25% of these patients will have recurrence of their disease. No tumour-associated marker to predict the recurrence of this subgroup of patients and define who could benefit from adjuvant therapy is used in clinical routine[1] In addition to immunoscore, other parameters of the immune response are investigated – mostly cellular immunity and the production of immunosuppressive and neoangiogenic markers. Vascular endothelial growth factor (VEGF) is the factor responsible for neoangiogenesis and it is being considered as a possible prognostic marker of disease progression. Transforming growth factor-beta (TGF-beta) is also neoangiogenic and a highly immunosuppressive factor as it suppresses the body's natural immunity against tumours and is also being considered as another possible prognostic marker of disease progression.

## Aim

To monitor the immune response in patients with stage II colorectal cancer, with a focus on cellular as well as humoral immunity. TGF-beta and VEGF levels were followed.

## Methods

38 patients with stage II colon cancer included in the research project received routine cancer treatment. . Basic parameters – histological type and grade, proliferative markers – were established at baseline. Patients were evaluated by a clinical immunooncologist to exclude any immune disorders of allergic or autoimmune origin. TGF-beta and VEGF were measured using ELISA, and anti-tumour cellular immunity (CD4, CD8, T-reg, B cells) were measured via flow cytometry.

## Results

In patients with stage II colorectal cancer, predominantly a depression in cellular immunity was seen. Plasma levels of immunglobulins were also reduced, particularly the IgG3 subtype. Most patients showed some clinical symptoms of immunodeficiency, such as frequent respiratory tract infections and/or herpetic infections. TGF-beta and VEGF plasma levels were increased.

## Conclusion

The correlation of these neoangiogenic and immunosuppressive factors, as well as the state of anticancer immunity, could help in the future as a prognostic marker and contribute to the selection of targeted adjuvant as well as immune therapy in patients with colorectal cancer stage II.

## Dedication

This project was supported by grant of Czech Ministery of Health, 15-28188A, League against cancer and PRVOUK.
